# *Candida albicans* Urinary Tract Infection with Suspected Fungal Hepatobiliary Disease in an Immunosuppressed Dog: A Case Report

**DOI:** 10.3390/ani16193136

**Published:** 2026-10-07

**Authors:** Amy Norman, Michelle Walton-Clark

**Affiliations:** 1 Linnaeus Veterinary Limited, Winchester SO21 2LL, UK; 2 Linnaeus Veterinary Limited, Edison Business Park, Swindon SN3 3FR, UK

**Keywords:** candiduria, cholangiohepatitis, fluconazole, opportunistic mycosis, hepatopathy, mycophenolate mofetil

## Abstract

Fungal urinary tract infections (UTIs) are uncommon in dogs and are typically associated with immunocompromised patients or those with other predisposing factors. Fungal hepatobiliary disease is rarely reported in the veterinary literature. This case report describes a dog on dual immunosuppressive therapy for suspected immune-mediated polyarthritis that developed a hepatopathy suspected to represent bacterial hepatobiliary disease and was subsequently treated with prolonged amoxicillin–clavulanate therapy. Following treatment, the dog developed marked hepatocellular enzyme elevations together with a *Candida albicans* UTI. Given the patient’s progressive hepatocellular injury, clinical signs, history of immunosuppression and antibiotic therapy, concurrent fungal hepatobiliary involvement was suspected. Following initiation of oral fluconazole, the fungal UTI resolved, and there was marked improvement, with subsequent normalisation of ALT and ALP activities. Although the temporal association between antifungal treatment and clinical and biochemical improvement raised suspicion of concurrent fungal hepatobiliary involvement, simultaneous discontinuation of amoxicillin–clavulanate, substantial reduction in prednisolone dose, and continuation of hepatoprotective therapy precluded attribution of the improvement specifically to antifungal treatment. This case highlights the potential for opportunistic fungal disease in immunosuppressed dogs, particularly following prolonged antimicrobial exposure, and illustrates the diagnostic challenges associated with suspected hepatobiliary involvement.

## Simple Summary

Fungal urinary tract infections are rare in dogs and are more likely to occur when there is an underlying predisposing factor or suppression of the immune system. This report describes a dog that was receiving medications to suppress the immune system, in addition to antibiotics for a prolonged period of time. The dog developed a *Candida albicans* infection in the bladder together with significant liver abnormalities, raising concern for possible fungal involvement of the liver. The dog was treated with fluconazole, an antifungal medication, and both the urinary tract infection and liver enzyme elevations resolved. Fungal involvement of the liver was never definitively proven. This case highlights that fungal infection involving more than one part of the body should be considered in dogs with relevant predisposing factors.

## 1. Introduction

Fungal urinary tract infections (UTIs) are uncommon in dogs, with *Candida* spp. accounting for less than 1% of positive canine urine cultures in one large retrospective study [1]. Reported predisposing factors include diabetes mellitus, immunosuppressive therapy, urinary catheterisation, lower urinary tract abnormalities, and previous antimicrobial administration [2,3,4]. *Candida albicans* is the most frequently isolated fungal organism, although other *Candida* species including *C. glabrata*, *C. tropicalis* and *C. parapsilosis* have also been reported [2,3].

*Candida albicans* is a commensal yeast that normally inhabits mucosal surfaces of the gastrointestinal, respiratory, and urogenital tracts of animals and humans [4]. Usually, host immune mechanisms and competition from normal bacterial microbiota limit fungal proliferation [4]. However, disruption of these protective mechanisms through immunosuppression, antimicrobial administration or concurrent disease may allow fungal overgrowth and tissue invasion [4,5]. Pathogenicity is facilitated by virulence factors such as adhesion to epithelial surfaces, biofilm formation, transition from yeast to hyphal forms, and production of hydrolytic enzymes that promote tissue invasion and dissemination [4,5,6,7].

Hepatic and hepatobiliary candidiasis are rarely reported in veterinary medicine [8,9,10,11,12,13,14,15]. Disseminated candidiasis has been described in dogs [8], and *Candida albicans* was identified within the gallbladder of a dog with concurrent diabetes mellitus, which was successfully managed with ketoconazole [9]. *Candida*-associated cholecystitis and hepatitis have been reported in one cat [10]. Additional reports have described hepatic mycosis occurring concurrently with fungal cystitis or as part of systemic candidiasis in dogs [8,11,12,13,14]. Haematogenous dissemination has been proposed as a possible route of hepatic involvement [8,12,13,14,15]. Despite these reports, hepatobiliary fungal disease remains poorly characterised in dogs, with little information regarding its diagnosis and management. Definitive diagnosis may be challenging because of low clinical suspicion, the need for invasive sampling, and difficulty obtaining histopathological or microbiological confirmation.

Currently, little information exists regarding concurrent urinary and hepatobiliary candidiasis in dogs, and there are no established treatment recommendations for cases with suspected concurrent urinary and hepatobiliary involvement [16]. The purpose of this report is to describe a *Candida albicans* UTI with suspected concurrent fungal hepatobiliary involvement in an immunosuppressed dog.

## 2. Case Presentation

A 3-year-old, male neutered crossbreed dog was presented to a referral hospital for investigation into a hepatopathy. Suspected primary immune-mediated polyarthritis (IMPA) had been diagnosed six months prior to referral. This was based on lethargy, shifting lameness, elevated C-reactive protein (CRP) (>200 mg/L; reference interval (RI) < 10) and response to treatment. Arthrocentesis was not performed to confirm the diagnosis. Immunosuppressive therapy was started with prednisolone at 1.1 mg/kg/day *per os* (PO), divided into two doses per day. Clinical response was monitored using CRP concentrations and clinical signs. Recurrence of clinical signs and increased CRP concentrations occurred on several occasions during attempts to reduce the prednisolone dose.

Routine monitoring prior to referral showed a mixed biochemical hepatopathy (27 and 10 weeks before referral; Table 1). Glucocorticoid-associated hepatopathy was thought to be most likely, and mycophenolate mofetil was started at 20.8 mg/kg/day PO, divided into two doses (13.9 mg/kg in the morning and 6.9 mg/kg in the evening) as a second immunosuppressive agent for poorly controlled suspected IMPA and as a steroid-sparing agent. Following the addition of mycophenolate and a decrease in prednisolone, the hepatocellular injury improved, but the cholestatic enzyme elevations worsened (as seen in Table 1, 8 and 6 weeks prior to referral). In addition, the patient had developed a progressive, regenerative anaemia, and he was referred for further investigations.

At the time of referral, the patient was reported to be progressively hyporexic and lethargic. He was receiving prednisolone at 1.1 mg/kg/day PO, divided into two doses, and mycophenolate mofetil at 20.8 mg/kg/day PO, divided into two doses. He had developed gastrointestinal signs with diarrhoea and melena. There had been a gradual decrease in haematocrit in addition to a mild reticulocytosis, consistent with gastrointestinal blood loss in the context of reported melena. On physical examination, the patient was bright, alert and responsive. He weighed 18 kg and had a body condition score (BCS) of 5/9. He had palpable hepatomegaly on abdominal palpation. The rest of his physical examination was within normal limits.

## 3. Initial Investigations

A full biochemistry profile was performed to assess the biochemical hepatopathy (week 0 in Table 2). Alanine aminotransferase (ALT) had decreased modestly to 476 U/L (RI 10–125 U/L), although marked cholestatic enzyme elevations persisted, with an alkaline phosphatase (ALP) of 3444 U/L (RI 23–212 U/L) and gamma-glutamyl transferase (GGT) of 410 U/L (RI 0–11 U/L). Aspartate aminotransferase (AST) was mildly increased at 85 U/L (RI 0–50 U/L). Hypercholesterolaemia remained severe at 10.77 mmol/L, and triglycerides were elevated at 2.74 mmol/L. Total bilirubin had returned to within the reference interval (RI 0–15 µmol/L).

Given the history of melena and the previously documented mild regenerative anaemia, haematology was performed. This revealed a moderate regenerative anaemia, characterised by a haematocrit of 25.7% (RI 37.3–61.7%) and reticulocytosis of 115.6 K/µL (RI 10–110 K/µL). A marked neutrophilia was also present at 24.68 × 10^9^/L (RI 2.95–11.64 × 10^9^/L).

An abdominal ultrasound was performed to assess the hepatobiliary system. This revealed severe, diffuse hepatomegaly and a moderately distended gallbladder containing anechoic bile and hyperechoic, rounded, mobile sediment with mild distal acoustic shadowing but no twinkling artefact. The gallbladder wall was mildly thickened and irregular, with no evidence of common bile duct distension. A splenic vein thrombus was present, with otherwise normal splenic architecture, and a small volume of free abdominal fluid was present. Partially mineralised sediment was present within the urinary bladder.

A urine sample was obtained to exclude a UTI, given his lethargy, polyuria (PU), and polydipsia (PD) at presentation. There was no evidence of bacterial or fungal urinary tract infection, and the bacterial culture was negative. The urine was poorly concentrated with a urine specific gravity (USG) of 1.014, and the UPC was increased at 0.9 (RI < 0.2). Poorly concentrated urine and proteinuria were considered most likely secondary to glucocorticoid administration [17,18].

Fine needle aspirates of the liver were obtained for cytology and culture. Cholecystocentesis was also performed, with bile submitted for culture. Cytology revealed non-lipidic vacuolation, likely reflecting hydropic change, together with mixed inflammation predominantly comprising neutrophils, which accounted for approximately 70% of the total nucleated cell count. Culture yielded *Staphylococcus epidermidis* from the liver enrichment broth, while bile culture was negative. Antimicrobial susceptibility testing indicated susceptibility to amoxicillin–clavulanate.

## 4. Initial Treatment

Although growth of *S. epidermidis* only in enrichment broth meant that contamination could not be excluded, it has been reported to be an opportunistic pathogen [19]. In this case, true infection was considered plausible given the recent dual immunosuppression, marked hepatopathy, predominantly neutrophilic mixed inflammation on hepatic cytology (neutrophils accounting for approximately 70% of the total nucleated cell count), and concurrent ultrasonographic hepatobiliary abnormalities. On this basis, the patient was treated for suspected bacterial hepatobiliary disease. The isolate was susceptible to amoxicillin–clavulanate, and amoxicillin–clavulanate was initiated at 20 mg/kg PO twice daily for a planned six-week course, consistent with reported antimicrobial treatment durations for canine bacterial hepatobiliary infections [20,21]. Given the gastrointestinal bleeding present at presentation, mycophenolate mofetil was discontinued, and the prednisolone dose was reduced to approximately 0.8 mg/kg/day PO, divided into two doses. S-adenosylmethionine and silybin (Denamarin, 425 mg PO once daily) were started for hepatoprotective support, ursodeoxycholic acid (UDCA; Destolit, 250 mg PO once daily) was started to support bile flow given that cholestatic disease was suspected, and omeprazole was administered at 1 mg/kg PO twice daily for suspected gastrointestinal ulceration.

Three weeks after initiation of treatment, the patient was reported to be much brighter. Biochemistry showed improvement in his ALP from 3444 U/L to 1931 U/L, although his ALT had increased to 777 U/L (week 3, Table 2). As the patient’s clinical signs and cholestatic enzyme elevations had improved, treatment was continued to complete the planned six-week course of amoxicillin–clavulanate.

## 5. Re-Evaluation

The patient was re-evaluated following completion of the antibiotic course, at which time there had been progression of his biochemical hepatopathy (shown in week 9 in Table 2). His ALT had increased from 777 U/L to 1544 U/L (RI 10–125 U/L) and his AST had increased from 85 U/L to 251 U/L (RI 0–50 U/L). Given the degree of hepatocellular injury, a bile acid stimulation test was performed, which demonstrated markedly increased pre- and post-prandial bile acid concentrations of 45.8 μmol/L and 121.8 μmol/L, respectively (RI 0–29.9 μmol/L), consistent with impaired hepatobiliary function and/or cholestasis. At this time, the patient was also demonstrating stranguria and pollakiuria, which had reportedly been present intermittently during treatment with prednisolone.

Repeat abdominal ultrasound showed largely unchanged hepatobiliary findings, and hepatic aspirates were repeated. The splenic vein thrombus was not specifically reassessed on subsequent imaging, and its outcome could not be determined. There was evidence of echogenic urinary sediment and urethral inflammation, and a high post-voiding residual volume was present within the bladder, raising suspicion for a functional outflow tract obstruction. Repeat liver cytology revealed similar changes to those previously. Repeat culture of the liver aspirates was negative.

Abdominal radiographs were obtained to assess for urolithiasis. No radiopaque uroliths were identified, and no structural abnormality was evident to explain the lower urinary tract signs. Radiolucent uroliths could not be excluded radiographically, although they were considered unlikely in the absence of supportive ultrasonographic findings. A reverse-contrast urethrocystogram would have been required for definitive exclusion.

Given the urinary signs, urinalysis and culture were performed on a cystocentesis urine sample. Urine was dark yellow and cloudy, with a USG of 1.013 and pH of 7.5. Dipstick analysis demonstrated 1+ protein and 1+ blood, with negative glucose, ketones and bilirubin. Sediment examination demonstrated 2–3 red blood cells/HPF, no white blood cells, casts, crystals, bacteria or epithelial cells. The UPC was 1.0 (RI < 0.2). In-house assessment of the urine sample revealed fungal hyphae and budding yeast. External sediment examination demonstrated 21–50 yeast/high-power field (HPF) (RI: none). For bacterial culture, urine was inoculated onto Columbia agar and MacConkey agar and incubated at 36 °C for 48 h. Fungal culture was performed on Sabouraud dextrose agar with chloramphenicol and actidone and incubated at 30 °C for three weeks. *Candida albicans* was identified using matrix-assisted laser desorption/ionisation time-of-flight mass spectrometry (MALDI-TOF). Fungal culture yielded a semi-quantitative result of 10,000–50,000 colony-forming units (CFU)/mL, based on the volume of urine inoculated and the proportion of the streaked area covered by colonies. Subsequent reports at two and three weeks of incubation identified no additional fungal growth beyond *C. albicans*. Antifungal susceptibility testing was not performed.

The biochemical pattern of the hepatopathy had changed substantially, with marked hepatocellular injury. Following diagnosis of the *C. albicans* urinary tract infection, concurrent hepatobiliary involvement was suspected. Liver biopsies were discussed but declined by the owner because of their invasive nature. Periodic acid–Schiff (PAS) staining was subsequently performed on the hepatic cytology specimens; however, the findings were inconclusive, and fungal invasion of the liver could neither be confirmed nor excluded.

## 6. Further Treatment

The identified *C. albicans* UTI was considered clinically significant given the concurrent urinary signs, and treatment was recommended. Given the marked increase in ALT, there was concern for possible concurrent fungal hepatobiliary involvement. The patient was therefore started on fluconazole at approximately 10 mg/kg (200 mg) PO once daily. Prazosin was started at 0.06 mg/kg PO twice daily to reduce urethral smooth muscle tone in view of the suspected functional outflow tract obstruction and high post-void residual bladder volume [22]. Denamarin and UDCA were also continued. The prednisolone dose was reduced to approximately 0.25 mg/kg/day PO. Given the potential for fluconazole-associated hepatotoxicity, serial biochemical monitoring was performed throughout treatment.

Twenty-two days after the initiation of fluconazole therapy, repeat urine cytology was negative for fungal organisms, fungal culture yielded no growth, and marked biochemical improvement was observed. ALT decreased from 1544 U/L to 222 U/L, while ALP decreased from 1980 U/L to 355 U/L (Table 2, week 13). The pollakiuria and stranguria resolved completely.

## 7. Outcome

The patient completed a 5-week course of fluconazole and underwent repeat biochemistry at the final follow-up (Table 2, week 17). This demonstrated normalisation of ALT and ALP activities, with an ALT of 106 U/L (RI 10–125 U/L) and an ALP of 173 U/L (RI 23–212 U/L). No repeat bile acid stimulation test or other specific assessment of hepatic function was performed; therefore, normalisation of liver enzyme activities should not be interpreted as documented recovery of hepatic function. Repeat urinalysis showed no evidence of fungal organisms on cytology or culture. The patient was clinically normal, with no recurrence of signs attributable to his suspected IMPA. Prednisolone was discontinued at this stage, along with UDCA and Denamarin.

## 8. Discussion

To the authors’ knowledge, detailed longitudinal reports describing the concurrent development of a confirmed *C. albicans* UTI and suspected hepatobiliary involvement, together with serial biochemical responses to systemic antifungal treatment, are limited in dogs. This case describes a dog with several recognised risk factors for opportunistic fungal infection, including dual immunosuppressive therapy and prolonged antimicrobial administration. Following treatment for suspected bacterial hepatobiliary disease, the initial cholestatic enzyme elevations improved; however, progressive hepatocellular injury subsequently developed.

Re-evaluation identified a clinically significant *Candida albicans* urinary tract infection concurrently with marked hepatocellular injury (ALT 1544 U/L; RI 10–125 U/L). Given the combination of immunosuppression, prolonged antimicrobial exposure, confirmed candiduria, and progressive hepatocellular injury, concurrent fungal involvement of the hepatobiliary system was suspected. Following initiation of systemic fluconazole therapy, the urinary tract infection resolved and there was rapid clinical and biochemical improvement. The temporal association between initiation of antifungal treatment and marked biochemical improvement raised suspicion of possible concurrent fungal hepatobiliary involvement, although a causal relationship could not be fully established.

This case had several possible predisposing factors for the development of opportunistic fungal disease. Glucocorticoids and mycophenolate mofetil were used concurrently for management of the suspected IMPA, likely causing significant immunosuppression. Immunosuppressive therapy is recognised as a risk factor for opportunistic fungal infection [2,5]. The patient also received a prolonged course of amoxicillin–clavulanate for suspected bacterial hepatobiliary disease, and antimicrobial administration has similarly been associated with an increased risk of opportunistic fungal infection [2,5]. The combination of prolonged immunosuppression and antimicrobial exposure likely created favourable conditions for fungal proliferation in the patient.

As a commensal organism of the gastrointestinal tract, *C. albicans* typically exists without causing disease [4]. However, disruption of host immunity and loss of microbial competition may allow transition from a commensal to an opportunistic infection [4,6,7]. In the present case, gastrointestinal ulceration was suspected based on the presence of melena. Gastrointestinal mucosal injury may have been associated with concurrent drug treatment, particularly mycophenolate mofetil, although a definitive cause was not established.

The route of fungal spread could not be determined in this case. Haematogenous dissemination was considered a possible mechanism for concurrent hepatobiliary involvement, particularly given the patient’s immunosuppressed state [8,12,13,14,15]. Translocation across the gastrointestinal mucosa may also have been possible, given the suspected gastrointestinal ulceration, antibiotic exposure, and consequent potential disruption of normal microbiota [23]. However, the urinary and hepatic abnormalities may also have represented separate processes, and the direction of any potential fungal dissemination could not be established.

This case demonstrated a confirmed *C. albicans* urinary tract infection based on urine culture and cytology, further supported by the presence of lower urinary tract signs, urethral inflammation, and a high post-voiding residual volume. Resolution of the clinical signs following fluconazole treatment, together with a negative repeat urine culture, provided further support for this diagnosis. In contrast, suspected fungal hepatobiliary involvement remained unconfirmed. Although the concurrent worsening biochemical hepatopathy, immunosuppression, antimicrobial exposure, and confirmed *C. albicans* UTI raised concern about fungal hepatobiliary involvement, fungal organisms were not demonstrated within the liver.

The treatment of this case presented a clinical challenge. Given the rarity of fungal UTIs in companion animals, there are currently no standardised treatment protocols [2,5,16]. Several case reports have described successful management using intravesical antifungal therapy with clotrimazole as well as systemic antifungal therapy [3,5,24]. Fluconazole reaches high levels in the urine due to its urinary excretion and is commonly recommended for the treatment of *Candida* UTIs in humans [25]. In the present case, the severe hepatocellular injury and multiple risk factors for opportunistic fungal disease raised concern for fungal hepatobiliary involvement in addition to the *C. albicans* UTI. Intravesical clotrimazole was considered as a local treatment option; however, this approach would only address the urinary tract infection and would not provide systemic therapy if concurrent fungal involvement of the hepatobiliary system was present [24]. Systemic fluconazole was therefore selected despite the potential for hepatotoxicity and the presence of progressive hepatocellular injury and evidence of impaired hepatobiliary function, including an abnormal bile acid stimulation test [26,27].

Fluconazole was administered at approximately 10 mg/kg once daily for 5 weeks, consistent with published veterinary dosing recommendations for urinary candidiasis [27,28]. Higher dosing regimens have been proposed for systemic fungal disease, including an initial dose of 10 mg/kg twice daily in dogs because of substantial interindividual variability in plasma fluconazole concentrations [26]. In the present case, hepatobiliary involvement was suspected but unconfirmed, and marked hepatocellular injury was already present. The once-daily regimen was based on published recommendations for the confirmed *C. albicans* UTI. Although systemic treatment was also desired because of concern for hepatobiliary involvement, the dose was not escalated to the higher twice-daily regimen in view of the pre-existing hepatocellular injury. Hepatic enzyme activities were monitored closely throughout treatment. Fluconazole was selected because it is considered one of the less hepatotoxic azole antifungals, achieves high urinary concentrations, and has good tissue penetration [4,25,27]. Although the potential for worsening hepatocellular injury was recognised, the concern for concurrent fungal hepatobiliary involvement was considered to outweigh this risk.

Twenty-two days after initiation of fluconazole therapy, there was marked biochemical improvement, with a reduction in ALT from 1544 U/L to 222 U/L (85.6% reduction) and ALP from 1980 U/L to 355 U/L (82.1% reduction). Concurrently, urine cytology showed no fungal organisms, fungal culture yielded no growth, and the patient’s lower urinary tract signs resolved. Although resolution of the lower urinary tract signs occurred following initiation of treatment, prazosin was commenced concurrently and may also have contributed to the clinical improvement. Microbiological clearance of *C. albicans* on repeat urine culture provided stronger evidence of response to antifungal therapy. The magnitude and rapidity of the biochemical improvement following initiation of antifungal therapy, occurring concurrently with resolution of the confirmed *C. albicans* UTI, strengthened the clinical suspicion of concurrent hepatobiliary involvement. However, in the absence of definitive identification of fungal organisms within the liver or biliary tract, this temporal association does not establish causality.

Alternative explanations for the progressive hepatopathy and subsequent biochemical improvement were also considered. Glucocorticoid-associated vacuolar hepatopathy may have contributed to the marked cholestatic enzyme elevations, particularly given the prolonged administration of immunosuppressive doses of prednisolone [29]. In dogs, glucocorticoid administration is typically associated with marked increases in ALP activity, including induction of the corticosteroid-associated ALP isoenzyme, whilst ALT activity increases are generally less pronounced. Although a glucocorticoid contribution could not be excluded, the subsequent progression of marked hepatocellular injury despite progressive reductions in the prednisolone dose suggested that glucocorticoid-associated hepatopathy alone was unlikely to explain the changing biochemical pattern.

Drug-induced hepatotoxicity was also considered. Mycophenolate mofetil had been discontinued at referral; however, amoxicillin–clavulanate was administered during the subsequent progression in hepatocellular injury and was discontinued at the time that fluconazole was initiated. Amoxicillin–clavulanate is a recognised cause of idiosyncratic drug-induced liver injury in people, while evidence in dogs is limited to isolated reports in which a causal association could not be definitively established [30,31]. Consequently, a contribution from drug-associated hepatotoxicity and/or biochemical improvement following withdrawal of amoxicillin–clavulanate could not be excluded.

Resolution or fluctuation of the previously suspected bacterial hepatobiliary disease could not be excluded, although repeat liver culture was negative, providing no evidence of persistent bacterial infection at that stage. Concurrent treatment with UDCA and S-adenosylmethionine may also have contributed to the subsequent biochemical improvement [32]. However, these hepatoprotective treatments had been initiated earlier in the disease course, during which hepatocellular injury continued to progress. Although the magnitude and timing of biochemical improvement following initiation of fluconazole were striking, the simultaneous alteration of other treatments prevents attribution of improvement specifically to antifungal treatment.

Several limitations should be considered in this case report. Most importantly, suspected fungal hepatobiliary involvement was not confirmed. Fungal organisms were not identified on hepatic cytology, repeat hepatic culture was negative, PAS staining was inconclusive, and liver biopsy for histopathology and tissue culture was not performed. In addition, several treatments were altered around the time fluconazole was initiated, including a reduction in prednisolone, while UDCA and S-adenosylmethionine were continued. Consequently, although the timing and magnitude of the biochemical response were supportive of possible fungal hepatobiliary involvement, a direct causal relationship between fluconazole treatment and the biochemical improvement could not be established.

## 9. Conclusions

This case report highlights the importance of considering opportunistic fungal infection in immunocompromised patients, particularly those that have received prolonged immunosuppressive or antimicrobial therapy. Although fungal hepatobiliary involvement was not confirmed in the patient, the concurrent *Candida albicans* urinary tract infection and progressive biochemical hepatopathy raised suspicion for concurrent fungal involvement of the hepatobiliary system. The subsequent clinical and biochemical improvement occurred following the initiation of fluconazole therapy, which may provide further support for suspected hepatobiliary fungal involvement; however, temporal association alone cannot establish causality. Concurrent discontinuation of amoxicillin–clavulanate, reduction in the prednisolone dose and continued hepatoprotective therapy represent important confounding factors. This case supports consideration of hepatobiliary fungal disease as a differential diagnosis in immunocompromised dogs with unexplained progressive hepatopathy, particularly when concurrent candiduria or fungal urinary tract infection is identified.

## Figures and Tables

**Table 1:** Pre-referral clinicopathological abnormalities relative to referral.

Parameter	-27 Weeks	-10 Weeks	-8 Weeks	-6 Weeks
ALT (10–125 U/L)	24	458	802	619
ALP (23–212 U/L)	203	1468	1940	>2000 *
GGT (0–11 U/L)	0	107	292	430
Total bilirubin (0–15 µmol/L)	4	3	8	19
HCT (37.3–61.7%)	42.5	39.0	35.3	28.8

* Value exceeded the upper reportable limit; dilution was not available.

**Table 2:** Post-referral clinical events and clinicopathological findings.

Parameter	Referral (Week 0)	Week 3	Week 9	Week 13	Week 17
Clinical event	Mycophenolate discontinued; prednisolone reduced; amoxicillin–clavulanate initiated	3 weeks of amoxicillin–clavulanate	Post amoxicillin–clavulanate; *C. albicans* UTI identified; fluconazole initiated 5 days later	22 days into fluconazole treatment; repeat fungal culture negative	Final follow-up
ALT (10–125 U/L)	476	777	1544	222	106
AST (0–50 U/L)	85	—	251	—	—
ALP (23–212 U/L)	3444	1931	1980	355	173
GGT (0–11 U/L)	410	279	329	—	—
Total bilirubin (0–15 µmol/L)	10	9	8	—	—
Bile acids (0–29.9 µmol/L)	—	—	Pre 45.8; post 121.8	—	—
HCT (37.3–61.7%)	25.7	32.3	34.9	—	—

## Data Availability

The data presented in this study are available on request from the corresponding author. The data are not publicly available due to privacy and ethical considerations relating to client-owned clinical case data.

## Abbreviations

ALP	Alkaline phosphatase
ALT	Alanine aminotransferase
AST	Aspartate aminotransferase
BCS	Body condition score
CFU	Colony-forming units
CRP	C-reactive protein
GGT	Gamma-glutamyl transferase
HCT	Haematocrit
HPF	High-power field
IMPA	Immune-mediated polyarthritis
PAS	Periodic acid–Schiff
PD	Polydipsia
PO	*Per os*
PU	Polyuria
RI	Reference interval
UDCA	Ursodeoxycholic acid
UPC	Urine protein:creatinine ratio
USG	Urine specific gravity
UTI	Urinary tract infection
